# Terminal–Edge–Cloud Collaborative Computation Offloading and Resource Allocation Strategy Based on Improved Mayfly Algorithm for District Heating Systems

**DOI:** 10.3390/s26103110

**Published:** 2026-05-14

**Authors:** Guo-Hong Chen, Hao-Yuan Ma, Wang Yu, Jing Wen, Ke Chen, Jia-Jian Wang, Shi-Dong Chen, Yun-Lei Sun

**Affiliations:** 1 School of Information and Electrical Engineering, Hangzhou City University, 51 Huzhou Street, Hangzhou 310015, China; chenguohong@hzcu.edu.cn (G.-H.C.); 2230201017@stu.hzcu.edu.cn (H.-Y.M.); yuwang200302@outlook.com (W.Y.); 32302066@stu.hzcu.edu.cn (J.W.); 2240201039@stu.hzcu.edu.cn (K.C.); 2250201024@stu.hzcu.edu.cn (J.-J.W.); 22302001@stu.hzcu.edu.cn (S.-D.C.); 2 School of Electrical and Automation Engineering, East China Jiaotong University, 808 Shuanggang East Street, Qingshanhu District, Nanchang 330013, China

**Keywords:** district heating systems, thermal internet of things, task offloading, resource allocation, Mixed-Integer Non-Linear Programming, improved mayfly algorithm, swarm intelligence

## Abstract

The rapid digitalization of district heating systems (DHSs) has driven the large-scale deployment of thermal Internet of Things (TIoT) sensors, which generate massive real-time operational data. Traditional centralized computing architectures struggle to process massive concurrent data. Furthermore, they fail to balance the stringent low-latency demands of real-time control tasks with the low-energy constraints of battery-powered terminal devices. To solve the complex problem of minimizing the weighted sum of system latency and energy consumption, we propose an Improved Mayfly Algorithm (IMA). The algorithm integrates five targeted structural enhancements: random position update masking, differential evolution (DE)-based crossover, targeted subset mutation with boundary scaling, adaptive population reset mechanism, and simulated annealing (SA)-driven local search, to efficiently navigate the high-dimensional rugged decision space and mitigate premature convergence. Extensive simulation results show that the proposed collaborative architecture achieves the lowest total system cost compared with traditional isolated computing paradigms (local-only, edge-only, and cloud-only). Notably, the proposed IMA reduces the total baseline weighted cost by 17.2% compared with the standard MA. Furthermore, under maximum practical industrial workloads (750 concurrent tasks, representing a highly complex 2250-dimensional MINLP space), the IMA maintains strong scalability and dominance, outperforming the second-best algorithm (BWO) by 15.8%. This research provides a low-latency, energy-efficient scheduling solution for TIoT-enabled DHS, and offers technical support for the intelligent and low-carbon transformation of urban energy infrastructure.

## 1. Introduction

In the context of the global transition toward low-carbon energy systems, district heating systems (DHSs) have become a critical component of urban energy infrastructure, as their operational efficiency directly affects both heating comfort for residents and the level of carbon emissions generated by cities. In practical DHS operation, high-frequency valve control and fault diagnosis tasks require end-to-end latency below 200 ms to ensure heating stability, while battery-powered thermal Internet of Things (TIoT) sensors need an average energy consumption below 0.1 W per hour to achieve a 2-year service life. However, traditional centralized computing architectures often bring latency exceeding 1.5 s and 3 × higher energy consumption than the constraint, which cannot meet the practical industrial requirements. Recent studies have emphasized that the digitalization and intelligent transformation of district heating networks are essential for improving system efficiency and enabling sustainable energy management [1,2]. In particular, the integration of advanced sensing technologies and intelligent control mechanisms is considered a key pathway toward the construction of next-generation zero-carbon thermal energy systems [3]. With the rapid development of information technologies, district heating networks are evolving from traditional centralized infrastructures into intelligent, data-driven cyber–physical systems capable of real-time monitoring, analysis, and control [1,2].

The widespread adoption of the thermal Internet of Things (TIoT) has further accelerated the intelligent transformation of DHS. Massive temperature, flow, and pressure sensors are deployed across heating substations, pipelines, and heat exchange units for real-time state monitoring, generating huge volumes of operational data for system optimization, fault diagnosis, and energy efficiency improvement [4,5]. However, traditional centralized cloud computing architectures struggle to process such massive data, often leading to severe transmission latency, network congestion, and excessive energy consumption. Consequently, edge computing has been recognized as an effective paradigm to process massive sensor data near the data sources, thereby reducing communication overhead and enabling low-latency data analytics in smart energy systems [6,7]. By bringing computational resources closer to sensing devices, edge computing allows time-critical applications such as fault detection, load prediction, and dynamic control in district heating systems to be performed more efficiently.

Despite these advantages, the deployment of edge computing in TIoT environments introduces new challenges related to resource allocation and task scheduling. Many edge devices deployed in district heating infrastructures operate under strict hardware constraints, including limited battery capacity, restricted computing capability, and limited communication bandwidth. If computational tasks generated by sensors are executed entirely on local devices, the limited resources may lead to excessive processing delay and rapid battery depletion. On the other hand, blindly offloading all tasks to edge servers may cause severe network congestion and resource contention. Therefore, designing efficient task offloading strategies that can balance computational delay and energy consumption has become a critical issue in edge-enabled smart heating systems.

In recent years, numerous studies have explored intelligent district heating technologies and data-driven optimization strategies. From the perspective of smart heating systems, several researchers have investigated digital twin-based approaches for modeling and optimizing district heating networks. Digital twin technologies enable the creation of virtual replicas of physical heating infrastructures, allowing real-time system monitoring and predictive analysis [8,9]. Other studies have focused on hydraulic balancing optimization and energy-efficient operation strategies for secondary heating networks, which can effectively improve heating performance and energy utilization efficiency [10]. Intelligent energy management strategies have also been proposed to enhance the operational flexibility of smart DHS [11]. However, these studies mainly focus on the physical layer optimization of heating systems, and rarely address the real-time data processing and computational resource scheduling challenges brought by massive TIoT sensors. These studies demonstrate that integrating digital technologies with district heating infrastructure is a promising direction for achieving intelligent and sustainable heating systems.

Meanwhile, edge computing has been widely investigated as a key enabling technology for large-scale Internet of Things applications. Early research introduced the concept of edge computing and highlighted its potential to address latency-sensitive applications and bandwidth limitations in distributed computing environments [12]. Subsequently, standardized frameworks for multi-access edge computing (MEC) were proposed to support the deployment of edge computing architectures in practical systems [13]. Extensive surveys have summarized the architecture and key technologies of edge computing systems, including task offloading models, resource allocation mechanisms, and application scenarios in IoT networks [7,14,15]. In the context of mobile edge computing, task offloading has become an important research topic because it enables computational tasks generated by resource-constrained devices to be partially or fully executed on nearby edge servers.

In the field of edge computing task offloading, Mach et al. systematically reviewed the architecture and fundamental mechanisms of mobile edge computing, laying the foundation for subsequent research on task offloading strategies [16]. Existing offloading methods mainly rely on heuristic algorithms, game-theoretic approaches, and machine learning techniques. For instance, Cen et al. proposed a combined algorithm based on potential game theory and particle swarm optimization (PSO) to solve multi-user task offloading problems in edge computing environments [17]. Han et al. developed a multi-objective adaptive deep reinforcement learning framework to achieve a balance between latency and energy consumption in distributed task offloading systems [18]. Similarly, Rasool et al. introduced an improved particle swarm optimization approach to address dynamic task scheduling under time-varying workloads [19]. Other studies have investigated delay-aware resource allocation strategies and adaptive offloading mechanisms for IoT-enabled smart city applications [20,21]. Although these approaches have demonstrated promising results, they often suffer from limitations such as high computational complexity or unstable performance under dynamic network conditions.

Meta-heuristic optimization algorithms have also attracted considerable attention in solving complex task offloading and resource allocation problems. Classical swarm intelligence algorithms, such as genetic algorithms and particle swarm optimization, have been widely applied in optimization problems due to their simplicity and global search capability. However, these algorithms may suffer from premature convergence and may easily become trapped in local optima when dealing with highly nonlinear optimization problems. To overcome these limitations, various nature-inspired meta-heuristic algorithms have been proposed in recent years, including grasshopper optimization algorithms, dung beetle optimization, and beluga whale optimization [22,23,24]. Among them, the Mayfly Algorithm (MA), proposed by Zervoudakis and Tsafarakis, integrates the evolutionary characteristics of swarm intelligence with mating behaviors observed in mayflies, enabling efficient exploration and exploitation of continuous optimization problems [25]. Due to its strong global search capability and fast convergence characteristics, the Mayfly Algorithm has been successfully applied to several complex optimization scenarios. For example, Sui et al. applied a multi-strategy fusion mayfly algorithm to task offloading and scheduling problems in IoT-based fog computing systems [26].

Despite the promising potential of meta-heuristic algorithms, integrating a Terminal–Edge–Cloud collaborative architecture into district heating systems presents a highly complex Mixed-Integer Non-Linear Programming (MINLP) problem. The joint optimization process involves strict coupling between discrete variables (i.e., offloading decisions among terminal, edge, and cloud) and continuous variables (i.e., communication bandwidth and CPU computing resource allocation proportions). This deep coupling, combined with non-smooth queuing delays, transforms the system’s fitness search space into a highly rugged and deceptive landscape. Consequently, standard heuristic algorithms, such as Particle Swarm Optimization (PSO) and the standard Mayfly Algorithm (MA), often struggle to balance between global exploration and local exploitation. They may suffer from premature convergence and become trapped in local optima, and may struggle to meet the stringent latency and energy requirements of high-concurrency thermal IoT networks.

Despite the above research progress, there are still several important research gaps. Existing research on intelligent DHS rarely considers the terminal–edge–cloud collaborative computing paradigm, and lacks a specialized offloading and resource allocation model tailored to the latency and energy constraints of TIoT scenarios. The task offloading and resource allocation problem in DHS is essentially a complex Mixed-Integer Non-Linear Programming (MINLP) problem with strong coupling between discrete and continuous variables, as well as non-smooth queuing delays. Classical heuristic algorithms (e.g., PSO, MA) suffer from premature convergence and local optima trapping when solving such problems, and cannot meet the stringent performance requirements of high-concurrency TIoT networks. Existing improved swarm intelligence algorithms for offloading problems lack a targeted design for the characteristics of DHS, and their robustness and scalability in high-concurrency industrial scenarios are insufficiently verified.

The main contributions of this work are summarized as follows:

1. Comprehensive System Modeling: This study constructs a rigorous terminal–edge–cloud collaborative computation offloading model tailored for TIoT-enabled DHS. Unlike generic offloading models, this work explicitly incorporates the unique characteristics of DHS, including step-characteristic queuing delays under edge concurrency constraints, chip-level energy consumption of resource-constrained terminals, and the quantitative trade-off between local, edge, and cloud execution modes.
2. Innovative Algorithmic Design (IMA): We propose an application-specific solver, the Improved Mayfly Algorithm (IMA), which tailors and integrates classical evolutionary mechanisms (such as DE and SA) to strictly map and resolve the discrete-continuous coupling and non-smooth queuing delays inherent in TIoT MINLP environments:
  - A random position update mask to restrict synchronous dimensional updates and prevent premature convergence.
  - An arithmetic crossover strategy based on Differential Evolution (DE) to dynamically balance discrete and continuous variable exploration.
  - A targeted subset mutation with boundary scaling to protect the superior genetic segments of elite individuals.
  - An adaptive population reset mechanism triggered by stagnation detection to effectively escape deep local optimum traps.
  - A Simulated Annealing (SA)-driven deep local search utilizing the Metropolis acceptance criterion, empowering the algorithm to jump out of deceptive local basins by probabilistically accepting inferior solutions.
3. Extensive Performance Validation: Comprehensive simulations are conducted under high-concurrency thermal IoT scenarios. The results demonstrate that the proposed IMA significantly outperforms state-of-the-art and classical benchmark algorithms, including Beluga Whale Optimization (BWO), Dung Beetle Optimizer (DBO), and PSO, exhibiting superior convergence accuracy, exceptional robustness, and minimized total system overhead.

The remainder of this paper is organized as follows. Section 2 introduces the system architecture and problem formulation of the thermal IoT-enabled district heating environment. Section 3 presents the proposed improved Mayfly Algorithm and describes its optimization mechanism in detail. Section 4 provides simulation results and performance evaluations of the proposed approach. Finally, Section 5 concludes the paper and discusses potential future research directions.

## 2. System Model and Problem Formulation

**Figure 1:**
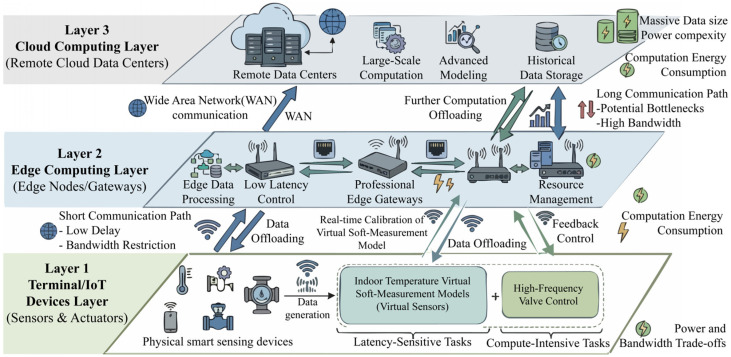
Terminal–Edge–Cloud collaborative computation offloading architecture diagram.

Modern District Heating Systems (DHS) integrate massive numbers of terminal devices. These IoT nodes generate exponential amounts of data. Some tasks, such as the real-time calibration of indoor temperature virtual soft-measurement models or high-frequency valve control, are highly latency-sensitive and compute-intensive. To address these challenges, this section establishes a rigorous Terminal–Edge–Cloud collaborative computation offloading model, as illustrated in Figure 1. This model accurately reflects the physical trade-offs between communication bottlenecks and computation energy consumption.

To clarify the applicable boundary of the proposed model and ensure the rigor of mathematical modeling, this paper makes the following basic assumptions for the terminal–edge–cloud collaborative offloading system in district heating scenarios:

(1) All computational tasks generated by thermal sensing terminals are indivisible atomic tasks. Each task can only be executed in one of the three exclusive modes: local computing, edge computing, or cloud computing, and partial offloading is not considered in this model. Atomic task assumption is adopted because the sensing and control tasks in DHS are usually small and indivisible, and partial offloading will bring additional data splitting and merging overhead, which is not suitable for resource-constrained TIoT terminals.
(2) The channel state information between terminal devices and the associated edge server is known and quasi-static. That is, the channel gain remains unchanged within a single scheduling cycle, and only changes across different scheduling cycles.
(3) All computational tasks arrive synchronously at the beginning of a scheduling cycle, and the dynamic random arrival of tasks during the scheduling cycle is not considered in this work.
(4) The central cloud platform has nearly unlimited computing resources. The internal queuing delay of cloud tasks is negligible compared with the fixed wide-area network propagation delay and cloud processing delay. Here, “nearly unlimited computing resources” refers to the fact that the central cloud platform has significantly more elastic and scalable computing capacity than edge servers within the considered scheduling horizon, rather than absolute infinite capacity. Therefore, its internal queuing delay is assumed to be negligible compared with the WAN transmission latency and the fixed cloud processing delay. This simplification is commonly adopted in edge-cloud offloading studies [15,26].
(5) The task execution process is non-preemptive. Once a task starts to be executed on the terminal, edge server, or cloud platform, it will not be interrupted by other tasks until the execution is completed.
(6) All terminal devices and edge servers work in a normal state during the scheduling cycle, and hardware failures, data packet loss, and retransmission caused by harsh thermal environments are not considered in the basic model.

### 2.1. Network Architecture

The proposed system adopts a three-tier collaborative architecture. It consists of a terminal layer with smart thermal sensors, an edge layer with aggregation gateways, and a central cloud computing layer. The system contains N edge servers. Each edge server covers M terminal devices. We define the set of concurrent tasks in a single scheduling cycle as I={1,2,…,N×M}.

Each task i∈I has specific computational attributes. We abstract these as a tuple $\left\langle T_{i},\;C_{i},\;f_{\mathrm{local}}\right\rangle$. Here, $T_{i}$ represents the input data size in bits. The parameter $C_{i}$ denotes the required CPU computation cycles to complete the task. The variable $f_{\mathrm{local}}$ is the inherent CPU clock frequency of the terminal device.

The system must determine the optimal execution path for each task. We define a discrete offloading decision variable $x_{i}\in \{1,2,3\}$. Specifically, $x_{i}=1$ indicates local execution at the terminal. The value $\;x_{i}=2\;$ signifies offloading to the edge server. The value $x_{i}=3$ denotes offloading to the cloud. Additionally, we define continuous variables $u_{i}\in \left[0,1\right]\;\mathrm{and}\;v_{i}\in \left[0,1\right]$. These represent the proportion of allocated uplink bandwidth and edge CPU computing resources for task i, respectively. Although real-world DHS involves stochastic tasks, we start with this deterministic baseline to address the fundamental MINLP complexity before transitioning to stochastic models in future work.

To facilitate the reader’s reference and ensure the consistency of mathematical symbols throughout the paper, all parameters, indices, physical meanings and units involved in the terminal–edge–cloud collaborative offloading model are systematically summarized in Table 1.

### 2.2. Communication Model

Tasks selected for non-local execution ($x_{i}\geq 2$) must transmit their data $T_{i}$ to the edge gateway via wireless channels. According to the Shannon–Hartley theorem, the uplink transmission rate $R_{i}$ for task i is formulated as:

(1) $$R_{i}=W\cdot u_{i}\cdot \log_{2}\left(1+\frac{P\cdot \eta_{i}}{\sigma^{2}}\right)$$

In this equation, W is the total channel bandwidth. The parameter P is the transmission power of the terminal device. The variable $\eta_{i}$ represents the channel state information, which includes path loss and antenna gain. The parameter $\sigma^{2}$ denotes the background noise power.

In practical high-concurrency heating IoT scenarios, edge servers have limited concurrent processing capabilities. We assume a maximum concurrency limit of MaxS. When task congestion occurs, the system employs a batch processing mechanism. Therefore, the actual transmission delay $D_{tx,i}$ includes both the physical transmission time and a step-characteristic queuing delay. Because tasks are processed in discrete batches bounded by the capacity limit MaxS, the waiting time exhibits a step-like function based on the task’s position in the queue, formulated as:

(2) $$D_{\mathrm{tx},i}=\frac{T_{i}}{R_{i}}+t_{\mathrm{wait}}^{\mathrm{tx}}\cdot \frac{{rank}_{i}}{MaxS}$$

Here, ${\mathrm{rank}}_{i}$ denotes the specific queuing order (or execution rank) of task i among all tasks currently offloaded to the associated edge server. The parameter $t_{\mathrm{wait}}^{tx}$ is the base waiting time for a single transmission round. This queuing delay model assumes that tasks arrive uniformly and are processed in batches according to the edge server’s maximum concurrent capacity *MaxS*, and the formula calculates the average waiting time of tasks in the transmission queue.

During the queuing period, the device remains in sleep or listening mode. This state does not consume significant transmission power. Thus, the terminal’s transmission energy consumption $E_{tx,i}$ depends solely on the active physical transmission time:

(3) $$E_{tx,i}=P\cdot \frac{T_{i}}{R_{i}}$$

### 2.3. Computation Model

The computational overhead of a task exhibits distinct physical characteristics based on the offloading decision $x_{i}$:

(1) Local Computing ($x_{i}=1$)

The task is processed locally at the thermal terminal. This mode eliminates transmission delays. However, the limited computing power of the terminal results in higher computation delay and consumes valuable battery life.

(4) $$D_{\mathrm{local},i}=\frac{C_{i}}{f_{\mathrm{local}}}$$

(5) $$E_{\mathrm{local},i}=K_{a}\cdot C_{i}\cdot {\left(f_{\mathrm{local}}\right)}^{2}$$

Here, $K_{a}$ represents the dynamic energy consumption coefficient of the local underlying chip.

(2) Edge Computing ($x_{i}=2$)

The task is executed on the edge server. Let the total computing capacity of the edge server be $C_{\mathrm{edge}}$. The actual operating frequency allocated to task i is $v_{i}\cdot C_{\mathrm{edge}}$. The total delay in this mode includes transmission delay, computation queuing delay, and actual computation delay:

(6) $$D_{\mathrm{edge},i}=D_{tx,i}+t_{\mathrm{wait}}^{cp}\cdot \left\lfloor \frac{{\mathrm{rank}}_{i}}{MaxS}\right\rfloor +\frac{C_{i}}{v_{i}\cdot C_{\mathrm{edge}}}$$

The total system energy consumption in the edge computing mode consists of the terminal’s transmission energy and the edge server’s computation energy. According to the CMOS circuit power dissipation laws, server energy consumption is proportional to the square of its operating clock frequency:

(7) $$E_{\mathrm{edge},i}=E_{tx,i}+K_{b}\cdot C_{i}\cdot {\left(v_{i}\cdot C_{\mathrm{edge}}\right)}^{2}$$

In this equation, $K_{b}$ is the inherent energy coefficient of the edge server.

(3) Cloud Computing ($x_{i}=3$)

The cloud offers nearly infinite computing resources but is physically distant. Tasks destined for the cloud must first be transmitted to the edge gateway before being forwarded across the Wide Area Network (WAN). Therefore, the total cloud computing delay comprises the initial transmission delay $D_{tx,i}$, the batch-processing queuing delay at the edge gateway prior to forwarding, and a fixed cloud processing and WAN propagation delay $t_{\mathrm{cloud}}$:

(8) $$D_{\mathrm{cloud},i}=D_{tx,i}+t_{\mathrm{wait}}^{cp}\cdot \left\lfloor \frac{{\mathrm{rank}}_{i}}{MaxS}\right\rfloor +t_{\mathrm{cloud}}$$

In this mode, the edge side does not bear the computation load. The only dynamic energy consumption recorded by the system is the terminal’s transmission energy $E_{tx,i}$.

### 2.4. Problem Formulation and Complexity Analysis

The core objective of this research is to jointly optimize the offloading decisions X, bandwidth allocations U, and computing resource allocations V. This optimization seeks the optimal balance between strict latency requirements and the lifespan of heating terminals.

We define the total delay (TotalDelay) and total energy consumption (TotalEnergy) of the system in a single scheduling cycle as:

(9) $$TotalDelay=\sum_{i\in I}\left(D_{\mathrm{local},i}\cdot \delta_{\left\{x_{i}=1\right\}}+D_{\mathrm{edge},i}\cdot \delta_{\left\{x_{i}=2\right\}}+D_{\mathrm{cloud},i}\cdot \delta_{\left\{x_{i}=3\right\}}\right)$$

(10) $$TotalEnergy=\sum_{i\in I}\left(E_{\mathrm{local},i}\cdot \delta_{\left\{x_{i}=1\right\}}+E_{\mathrm{edge},i}\cdot \delta_{\left\{x_{i}=2\right\}}+E_{\mathrm{tx},i}\cdot \delta_{\left\{x_{i}=3\right\}}\right)$$

To comprehensively evaluate the system benefits, we construct a weighted cost function F. There is a massive numerical gap between latency (in seconds) and energy consumption (in Joules), often spanning several orders of magnitude. Therefore, we must introduce a physical dimension conversion constant $\mu =3.6\times 10^{6}$. Note that the parameter $\mu =3.6\times 10^{6}$ in Equation (11) is not an empirical tuning weight, but a strict physical dimension conversion constant. In DHS, calculating physical energy consumption typically yields values in millions of Joules, while latency is measured in seconds. To prevent the optimization cost function from being mathematically dominated by the massive numerical scale of the energy term, μ exactly converts Joules into standard industrial Kilowatt-hours (kWh), allowing the algorithm to optimize their trade-off without bias. This constantly converts Joules to kilowatt-hours to prevent the optimization process from becoming ineffective:

(11) $$F=w_{1}\cdot TotalDelay+w_{2}\cdot \frac{TotalEnergy}{\mu }$$

(12) $$s.t.x_{i}\in \{1,2,3\},\forall i\in I$$

(13) $$\sum_{i\in I_{edge}}u_{i}\leq 1,\sum_{i\in I_{edge}}v_{i}\leq 1$$

The proposed model reveals that the cloud-edge resource scheduling in heating systems is a highly complex Mixed-Integer Non-Linear Programming (MINLP) problem, which has been proven to be NP-hard. The discrete decision variables (offloading locations) are deeply coupled with continuous decision variables (resource allocation proportions). Furthermore, the queuing delay function (involving a floor operation) introduces non-smooth step characteristics. Consequently, the fitness search space of the system is extremely “rugged” and riddled with deceptive local optima. Due to the strong coupling between discrete and continuous variables, non-smooth stepwise queuing delay, and non-convex feasible domain, traditional gradient-based optimization methods are inapplicable to this problem, while simple heuristic algorithms suffer from severe premature convergence and cannot find the global optimal solution. This motivates us to design a tailored, improved mayfly algorithm to solve this complex MINLP problem efficiently.

## 3. Proposed Improved Mayfly Algorithm (IMA)

The joint optimization of offloading decisions and resource allocation is an NP-hard Mixed-Integer Non-Linear Programming (MINLP) problem. To solve this complex problem, we propose the Improved Mayfly Algorithm (IMA). The IMA incorporates five core evolutionary mechanisms to overcome the rugged and deceptive fitness landscape of the thermal Internet of Things (IoT) system. Unlike conventional adaptations that focus entirely on the continuous dynamic adjustment of weight coefficients, our IMA strictly maps classical mechanisms to resolve deep discrete-continuous coupling [25]. Specifically, we integrate Differential Evolution (DE) crossover to maintain population diversity within the discrete task-offloading decision space, Simulated Annealing (SA) to enhance local exploitation for continuous resource variables, and a stagnation-triggered reset mechanism. These structural enhancements are tailored to handle the highly heterogeneous sensing workloads and strict physical boundary constraints of District Heating Systems (DHS).

### 3.1. Solution Representation and Encoding Strategy

The IMA adopts a unified encoding scheme to handle both discrete and continuous variables simultaneously, where each mayfly individual corresponds to a feasible scheduling solution. Specifically, the proposed approach encodes the position of each individual as a one-dimensional real-number array with a length of 3×N×M, and the values of the array are strictly constrained within the interval $\left[0,1\right]$. Mapping continuous variables [0, 1] to discrete offloading decisions {1, 2, 3} via the rounding function inherently creates a step-function fitness landscape with zero-gradient plateaus, which typically causes gradient-based algorithms to fail. To address this, the IMA introduces Differential Evolution (DE)-based crossover and Targeted Subset Mutation, enabling the algorithm to make probabilistic jumps across discrete boundaries without being trapped in flat regions. Furthermore, the resource allocation constraints defined in Equation (13) are strictly enforced through a dynamic normalization mechanism. The raw continuous values generated by the mayfly individuals are dynamically normalized by their sum, inherently guaranteeing 100% boundary feasibility without the computational overhead of penalty functions.

Logically, the position array is divided into three segments:

- Offloading Decision Segment: Continuous values are mapped to discrete integers {1,2,3} via the rounding function $Selo=\mathrm{round}\left(X_{i}\times 2\right)+1$, where integers 1, 2, and 3 respectively indicate that a task is executed locally, at the edge, or in the cloud.
- Transmission Resource Segment: This segment is used to determine the uplink bandwidth allocation. The exact bandwidth proportion for edge-bound tasks is calculated through value normalization.
- Computing Resource Segment: This segment is employed to control the CPU cycle allocation, and the precise proportion of edge computing resources is assigned via value normalization.

### 3.2. Overview of the Standard Mayfly Algorithm (SMA)

The standard Mayfly Algorithm (MA) is a swarm intelligence algorithm inspired by the flight and mating behaviors of mayflies. Specifically, male mayflies adjust their movement trajectories based on their personal best position (pbest) and the global best position (gbest). In contrast, female mayflies do not form swarms; instead, they update their velocities to move toward male mayflies for reproduction.

Male mayflies are the dominant group of the population, and they will adjust their flight trajectory according to their own historical optimal position (pbest) and the global optimal position of the population (gbest). The velocity and position update formulas are:

(14) $$v_{i}^{t+1}=v_{i}^{t}+a_{1}\cdot e^{-\beta r_{p}^{2}}\cdot \left(pbest_{i}-x_{i}^{t}\right)+a_{2}\cdot e^{-\beta r_{g}^{2}}\cdot \left(gbest-x_{i}^{t}\right)$$

(15) $$x_{i}^{t+1}=x_{i}^{t}+v_{i}^{t+1}$$

where $v_{i}^{t}$ and $x_{i}^{t}$ represent the velocity and position of the i-th male mayfly at the t-th iteration, respectively; $a_{1}$ and $a_{2}$ are the positive attraction coefficients of individual experience and global experience, respectively; β is the visibility coefficient controlling the attenuation of attraction; $r_{p}\;\mathrm{and}\;r_{g}$ are the Euclidean distances between the current individual and its pbest, and between the current individual and gbest, respectively. For male individuals with poor fitness, the algorithm will introduce a random flight mechanism, and its velocity update formula is:

(16) $$v_{i}^{t+1}=v_{i}^{t}+d\cdot r_{\mathrm{rand}}$$

where d is the dance coefficient, and $r_{rand}$ is a random number obeying the standard normal distribution.

Female mayflies will fly towards male mayflies for courtship and mating, and their velocity update follows the attraction rule based on the fitness of male individuals. The velocity and position update formulas are:

(17) $$v_{i}^{t+1}=\left\{\begin{array}{c} v_{i}^{t}+a_{3}\cdot e^{-\beta r_{m}^{2}}\cdot (x_{\mathrm{male},i}^{t}-x_{i}^{t}),\;f(x_{i}^{t})>f(x_{\mathrm{male},i}^{t})\\ v_{i}^{t}+fl\cdot r_{\mathrm{rand}},\;otherwise \end{array}\right.$$

(18) $$x_{i}^{t+1}=x_{i}^{t}+v_{i}^{t+1}$$

where $a_{3}$ is the attraction coefficient of female individuals; $r_{m}$ is the Euclidean distance between the female individual and the corresponding male individual; fl is the random flight coefficient of female individuals; f is the fitness function of the optimization problem.

The algorithm selects male and female parents according to the fitness ranking, and generates offspring through crossover operation. The offspring’s position is calculated by:

(19) $$\mathrm{of}f_{1}=L\cdot x_{\mathrm{male}}+\left(1-L\right)\cdot x_{\mathrm{female}}$$

(20) $$\mathrm{of}f_{2}=L\cdot x_{\mathrm{female}}+\left(1-L\right)\cdot x_{\mathrm{male}}$$

where L is a random number obeying uniform distribution.

However, the standard MA has limitations in handling high-dimensional MINLP problems and is prone to premature convergence when navigating highly rugged search spaces.

### 3.3. Random Position Update Mask

The standard MA adopts full-dimensional synchronous position update, which will simultaneously modify the discrete offloading decision and continuous resource allocation variables of the same task. For the DHS offloading problem, this will destroy the excellent offloading decision that has been found, leading to premature convergence. To address this issue, we introduce a random position update mask mechanism to realize probabilistic selective updating of position dimensions. Specifically, the IMA abandons the synchronous full-dimensional update strategy and selectively updates position dimensions based on a preset probability.

The algorithm generates a binary mask vector $M_{\mathrm{rand}}$ using the condition $\mathrm{rand}\left(1,nVar\right)<rand\times 0.5$, and the position update is completed through the Hadamard product, as shown in the following formula:

(21) $$x_{i}^{t+1}=x_{i}^{t}+\left(v_{i}^{t+1}⊙M_{\mathrm{rand}}\right)$$

This position update logic is applicable to all male and female mayfly individuals. Such a probabilistic dimensional constraint can avoid destructive genetic updates and thus effectively prevent premature convergence.

### 3.4. Arithmetic Crossover Strategy Based on Differential Evolution

Offspring are generated from selected male and female parents during the mating process. To address the mixed-variable nature of the thermal IoT offloading problem, the IMA adopts a hybrid crossover strategy.

In the mating phase, the algorithm triggers an arithmetic crossover inspired by Differential Evolution with a 50% probability. Two offspring are generated using a uniformly distributed random vector L, which is formulated in Equations (19) and (20).

If the probability threshold is not met, the algorithm seamlessly reverts to the standard shuffle crossover. This dual-track strategy can dynamically balance the exploitation capability of continuous resource variables and the exploration capability of discrete offloading decisions.

### 3.5. Targeted Subset Mutation with Boundary Scaling

The standard SMA adopts fixed-range mutation, which lacks flexibility. To maintain population vitality and avoid excessive perturbation, the IMA employs a targeted subset mutation strategy.

With a 50% probability, the algorithm randomly selects a subset of variable dimensions (specifically, 1% of the total dimensions) for perturbation. Furthermore, the mutation step size σ is strictly scaled to 10% of the search space boundaries, which is calculated as follows:

(22) $$\sigma_{j}=0.1\cdot \left(UB_{j}-LB_{j}\right)$$

(23) $$x_{\mathrm{mutant},j}=x_{j}+\sigma_{j}\cdot N\left(0,1\right)$$

This targeted perturbation can avoid the destruction of superior genetic segments of elite individuals caused by excessive step sizes, thereby maintaining the overall quality of the population.

### 3.6. Stagnation Detection and Adaptive Population Reset

A large number of deceptive local optima exist in the solution space of the MINLP offloading problem, which easily leads to algorithm stagnation. To address this issue, the IMA introduces an elite-preserving global reset mechanism.

The algorithm monitors the improvement of the global best fitness in real time. If the improvement magnitude is less than $10^{-5}$, a stagnation counter is incremented. When the counter exceeds 10% of the maximum number of iterations, a population reset is triggered: the historical best solution is strictly preserved, while all other non-elite male and female mayflies are completely re-initialized. This population re-initialization operation forces the algorithm to escape local optimum traps and explore entirely new regions of the solution space.

### 3.7. Simulated Annealing-Driven Deep Local Search

The standard SMA only relies on basic population evolution operations, lacking deep local search capability for the rugged fitness landscape of the MINLP problem. It is easy to fall into deep local optima in the late iteration stage and cannot further improve the convergence accuracy. To solve this problem, we introduce a simulated annealing (SA)-driven deep local search module at the end of each iteration loop, which mitigates local optima stagnation by applying the Metropolis criterion to probabilistically accept sub-optimal candidate solutions.

Each male mayfly has a 10% probability of entering a Markov chain loop, where the individual undergoes Gentimes iterations of local Gaussian mutation.

Specifically, a new candidate solution $x_{\mathrm{new}}$ is generated by adding a temperature-dependent Gaussian perturbation Δx to the current solution $x_{\mathrm{current}}$:

(24) $$x_{\mathrm{new}}=x_{\mathrm{current}}+\Delta x,\;\Delta x∼N\left(0,\sigma_{\mathrm{temp}}^{2}\right)$$

where the standard deviation $\sigma_{temp}$ of the Gaussian distribution scales dynamically with the current system temperature Temp and the search space boundaries (UB and LB):

(25) $$\sigma_{\mathrm{temp}}=\mathrm{Temp}\cdot \left(UB-LB\right)$$

The newly generated solution is evaluated using the Metropolis acceptance criterion, which is defined as follows:

(26) $$P_{\mathrm{accept}}=\left\{\begin{array}{c} 1,\;F_{\mathrm{new}}<F_{\mathrm{current}}\\ \exp\left(\frac{F_{\mathrm{current}}-F_{\mathrm{new}}}{\mathrm{Temp}}\right)>\mathrm{rand},\;F_{\mathrm{new}}\geq F_{\mathrm{current}} \end{array}\right.$$

The system temperature Temp decays exponentially according to the present cooling rate (set to 0.95 in this study). This thermal dynamic enables the algorithm to probabilistically accept inferior solutions, thereby endowing the IMA with strong robustness in breaking through the rugged barriers of the MINLP search space and finding the true global optimal solution.

### 3.8. Summary of the IMA Framework

The complete execution flow of the proposed Improved Mayfly Algorithm (IMA) is presented in Algorithm 1, which integrates all five core improvement mechanisms proposed in this paper.
**Algorithm 1** Improved Mayfly Algorithm (IMA)Input: Population size PopSize, maximum iterations IterMax, initial temperature $T_{0}$, cooling rate α, SA inner loop Gentimes, and algorithm parameters ($g,a_{1},a_{2},a_{3},\beta ,d,fl$). Output: The global optimal solution $x_{\mathrm{gbest}}$ and its fitness value $F_{\mathrm{gbest}}$.1: Initialize male population M and female population F with size PopSize randomly. 2: Evaluate fitness $F\left(x\right)$ for all individuals. 3: Initialize personal best $x_{\mathrm{pbest}}$ and global best $x_{\mathrm{gbest}}$. 4: Initialize current temperature $T=T_{0}$, and stagnation counter $C_{\mathrm{stag}}=0$. 5: **while** Iter<IterMax **do** 6:   **for** each female i ∈{1,2,…,PopSize} **do** 7:    Generate stochastic search mask $m_{\mathrm{search}}\leftarrow \mathrm{rand}\left(1,D\right)<\mathrm{rand}\times 0.5$ [*Improvement 1: Stochastic Mask*] 8:    Update female velocity $v_{f,i}$ and position $x_{f,i}$ restricted by $m_{\mathrm{search}}$. 9: **end for** 10: **for** each male i∈{1,2,…,PopSize} **do** 11:   Generate stochastic search mask $m_{\mathrm{search}}\leftarrow \mathrm{rand}\left(1,D\right)<\mathrm{rand}\times 0.5$ [*Improvement 1: Stochastic Mask*] 12:   Update male velocity $v_{m,i}$ towards $x_{\mathrm{pbest}}$ and $x_{\mathrm{gbest}}$, or execute Nuptial Dance. 13:   Update male position $x_{m,i}$ restricted by $m_{\mathrm{search}}$. 14:   Evaluate fitness and update $x_{\mathrm{pbest},i}$ and $x_{\mathrm{gbest}}$. 15: **end for** 16: Rank M and F in descending order of fitness. 17: **for** k = 1 to PopSize/2 **do**
18:   Select ranked parents to mate. 19: [*Improvement 2: Hybrid Crossover*] 20: **if** rand < 0.5 **then** Apply DE-based Arithmetic Crossover. 21: **else** Apply Shuffle Crossover. 22: **end for** 23: **for** k=1 to $N_{\mathrm{mutant}}$ **do**
24:   [*Improvement 3: Hybrid Mutation*] 25:   **if** rand < 0.5 **then** Apply Large-step Gaussian Mutation. 26:   **else** Apply Standard Gaussian Mutation. 27: **end for** 28: Merge parents, offspring, and mutants, then select the top PopSize males and females. 29: [*Improvement 4: Stagnation Escape Mechanism*] 30: **if** $\left|F\left(x_{\mathrm{gbest}}^{t}\right)-F\left(x_{\mathrm{gbest}}^{t-1}\right)\right|<10^{-5}$ **then** $C_{\mathrm{stag}}=C_{\mathrm{stag}}+1$ 31: **if** $C_{\mathrm{stag}}>0.1\times \mathrm{IterMax}$ **then**
32:   Retain the best male and female; randomly reinitialize the rest of the population. 33:   $C_{\mathrm{stag}}=0$
34: **end if**
35: [*Improvement 5: Simulated Annealing Local Search*] 36: **for** each male i∈{1,2,…,PopSize} **do** 37:   **if** rand < 0.1 **then** 38:    **for** s=1 to Gentimes **do** 39:     Generate neighborhood solution $x_{new}$ via Gaussian mutation. 40:     **if** $F\left(x_{\mathrm{new}}\right)>F\left(fx_{m,i}\right)$ **or** $\;exp[(F\left(x_{new}\right)-F_{\mathrm{gbest}})/T]>\mathrm{rand}$ **then** 41:     Accept $x_{\mathrm{new}}$ as $x_{m,i}$. 42:     Update $x_{\mathrm{pbest},i}$ and $x_{\mathrm{gbest}}$ if necessary. 43:     **end if**
44:    **end for**
45:   **end if**
46: **end for** 47: Dampen algorithmic coefficients (g, dance, fl). 48: Cool down temperature: T=T×α. 49: Iter=Iter+1 50: **end while** 51: **return** $x_{\mathrm{gbest}}$ and $F_{\mathrm{gbest}}$


To clarify the implementation logic of each step and pinpoint the exact positions of the five core improvements in the algorithm flow, a detailed line-by-line explanation corresponding to Algorithm 1 is provided in Table 2. From a computational perspective, the complexity of the proposed IMA is mainly dominated by population updating, fitness evaluation, crossover/mutation operations, and the SA-based local search. Let P denote the population size of each gender, D=3×N×M denote the dimension of each individual, $T_{\max}$ denote the maximum number of iterations, and $G_{\mathrm{SA}}$ denote the number of inner SA search steps. In each iteration, the update, decoding, and fitness evaluation procedures require approximately O(PD) operations, while sorting and mating introduce an additional O(P log P) cost. The SA-driven local search further contributes $O(p_{\mathrm{SA}}PG_{\mathrm{SA}}D)$, where $p_{\mathrm{SA}}$ is the probability that a male mayfly enters the SA procedure. Therefore, the overall computational complexity of the IMA can be expressed as

(27) $$O\left(T_{\max}\left(PD+P\log P+p_{\mathrm{SA}}PG_{\mathrm{SA}}D\right)\right).$$

Since D is typically much larger than log P in the considered DHS offloading scenario, the complexity is mainly dominated by the population evolution and local search terms, which can be further simplified as

(28) $$O\left(T_{\max}\cdot P\cdot D\left(1+p_{\mathrm{SA}}G_{\mathrm{SA}}\right)\right).$$

**Table 2:** Detailed Line-by-Line Explanation of Algorithm 1.

Line(s)	Explanation
Input	Defines all hyperparameters required for the algorithm execution, including population size, maximum iterations, simulated annealing (SA) parameters, and basic swarm intelligence coefficients.
Output	Specifies the final output of the algorithm: the global optimal scheduling solution and its corresponding minimum weighted cost value.
1	Initializes the male and female mayfly populations with the specified size PopSize. Each individual is represented as a 3×N×M-dimensional real-number array with values constrained to [0, 1], corresponding to offloading decisions, bandwidth allocations, and computing resource allocations respectively.
2	Evaluates the fitness value (weighted system cost F) for all initialized individuals using the objective function defined in Equation (11).
3	Initializes the personal best position (pbest) for each individual (initially its own position) and the global best position (gbest) for the entire population (initially the individual with the lowest fitness value).
4	Initializes the initial temperature T for the SA module and the stagnation counter $C_{stag}$(used to detect algorithm convergence stagnation).
5	Starts the main iteration loop of the algorithm, which runs until the maximum number of iterations IterMax is reached.
6–9	Updates the velocity and position of all female mayfly individuals: - Line 7: Improvement 1 (Random Position Update Mask): Generates a binary stochastic search mask $m_{search}$ where each dimension has a 50% probability of being updated, avoiding full-dimensional synchronous updates that destroy excellent genetic segments. - Line 8: Updates female velocity according to Equation (17) (attracted to superior males or performs random flight), then updates position restricted by the mask using Equation (21).
10–15	Updates the velocity and position of all male mayfly individuals: - Line 11: Applies the same random position update mask as female individuals (Improvement 1). - Line 12: Updates male velocity according to Equation (14) (towards pbest and gbest) or performs nuptial dance (Equation (16)) for inferior individuals. - Line 13: Updates male position restricted by the mask using Equation (21). - Line 14: Re-evaluates fitness and updates pbest for each male and the global gbest if a better solution is found.
16	Ranks male and female populations in descending order of fitness (lower cost = better fitness) to prepare for parent selection in the mating phase.
17–22	Performs mating and offspring generation: - Line 18: Selects the top PopSize/2 males and females as parents based on fitness ranking. - Line 20–21: Improvement 2 (DE-based Arithmetic Crossover): With 50% probability, applies differential evolution-inspired arithmetic crossover (Equations (19) and (20)) to generate offspring, which balances exploration of discrete offloading decisions and exploitation of continuous resource variables. - Line 21: With 50% probability, reverts to standard shuffle crossover to maintain population diversity.
23–27	Performs mutation operation on the offspring population: - Line 25–26: Improvement 3 (Targeted Subset Mutation with Boundary Scaling): With 50% probability, randomly selects 1% of the total dimensions for perturbation with a step size scaled to 10% of the search space boundary (Equations (22) and (23)), avoiding excessive destruction of elite genetic segments. With 50% probability, applies standard Gaussian mutation.
28	Merges the parent population, offspring population, and mutant individuals, then selects the top PopSize males and females with the best fitness to form the new population for the next iteration.
29–34	Implements the stagnation detection and adaptive population reset mechanism: - Line 30: Checks if the improvement of the global best fitness is less than $10^{-5};$ if so, increments the stagnation counter. - Line 31–34: Improvement 4 (Adaptive Population Reset): If the stagnation counter exceeds 10% of the maximum iterations, retains only the best male and female individuals and randomly reinitializes all other individuals to escape deep local optimum traps, then resets the stagnation counter.
35–46	Implements the simulated annealing-driven deep local search module: - Line 37: Each male individual has a 10% probability of entering the SA local search loop. - Line 38–44: Improvement 5 (SA-driven Local Search): Performs Gentimes iterations of temperature-dependent Gaussian mutation (Equations (24) and (25)) to generate neighborhood solutions. Accepts new solutions using the Metropolis criterion (Equation (26)), which probabilistically accepts inferior solutions to escape deceptive local basins. Updates pbest and gbest if a better solution is found.
47	Dampens the algorithmic coefficients (attraction coefficients, dance coefficient, random flight coefficient) to gradually shift the algorithm from global exploration to local exploitation as iterations progress.
48	Cools down the system temperature exponentially according to the cooling rate α=0.95, reducing the probability of accepting inferior solutions in the SA module in later iterations.
49	Increments the iteration counter.
50	Ends the main iteration loop.
51	Returns the global optimal solution $x_{gbest}$ and its corresponding minimum fitness value $F_{gbest}.$

Although the proposed enhancements increase the per-iteration computational cost compared with the standard MA, they significantly improve search robustness and solution quality for the mixed discrete-continuous MINLP problem.

In summary, the proposed IMA framework tightly integrates spatial exploration, targeted exploitation, and robust local optima escape mechanisms to effectively tackle the MINLP computation offloading problem. By mapping the discrete offloading decisions and continuous resource allocation proportions into a unified one-dimensional real-number encoding structure, the IMA successfully bridges the physical thermal IoT system with the mathematical optimization space. The synergistic operation of the random position update mask, DE-based arithmetic crossover, targeted subset mutation, adaptive stagnation reset, and SA-driven deep local search guarantees a high probability of finding the true global optimum while avoiding premature convergence and stagnation. This advanced scheduling framework not only addresses the limitations of the standard SMA in handling mixed-variable optimization problems but also establishes a solid theoretical foundation for the subsequent performance validation in highly concurrent district heating IoT environments.

## 4. Simulation Results and Performance Evaluation

To comprehensively verify the effectiveness and superiority of the proposed Terminal–Edge–Cloud collaborative strategy and the Improved Mayfly Algorithm (IMA), extensive simulation experiments are conducted in this section. The simulations are implemented using MATLAB R2022b on a standard computing platform equipped with an Intel Core i5 processor and 16 GB of RAM. The performance evaluation is structurally divided into five subsections: superiority analysis of the collaborative architecture, convergence comparison with state-of-the-art algorithms, statistical robustness assessment, ablation study of the IMA mechanisms, and scalability analysis under dynamic task concurrency.

### 4.1. Simulation Environment and Parameter Settings

The simulated physical scenario is established based on a high-concurrency thermal Internet of Things (IoT) environment in a district heating system. The system consists of N = 5 edge servers (aggregation gateways), and each edge server provides coverage for M = 100 terminal devices (e.g., smart temperature and pressure sensors). In a single scheduling cycle, the input data size $T_{i}$ and the required CPU cycles $C_{i}$ for each sensing task are randomly generated within specific intervals to mimic real-world heterogeneous thermal data requests. To balance the massive numerical gap between latency (in seconds) and energy consumption (in Joules), the weighted coefficients in the cost function are strictly set. The detailed physical and network parameters of the thermal IoT system are summarized in Table 3.

The physical and network parameters listed in Table 3 are strictly calibrated against mainstream industrial hardware specifications and widely accepted baselines in the edge computing literature [27,28,29]. Specifically, the terminal CPU frequency (1.1 GHz) and transmission power (0.25 W) reflect the typical characteristics of widely deployed low-power industrial IoT microprocessors (e.g., ARM Cortex-A series). The edge server computing capacity (20 GHz) and channel bandwidth (50 MHz) mirror typical multi-core industrial edge gateways and standard industrial WLAN protocols, respectively. Moreover, the wide intervals for input data size (0.5–20 MB) and required CPU cycles (0.1–6 × 10^9^) are specifically designed to encompass the highly heterogeneous sensing workloads in real DHS, ranging from lightweight scalar telemetry to compute-intensive thermal imaging tasks.

For the algorithmic performance comparison, the proposed IMA is benchmarked against six state-of-the-art and classical heuristic algorithms: Beluga Whale Optimization (BWO) [23], Particle Swarm Optimization (PSO) [30], standard Mayfly Algorithm (MA) [25], Chicken Swarm Optimization (CSO) [31], Gazelle Optimization Algorithm (GOA) [32], and Dung Beetle Optimizer (DBO) [22]. To ensure absolute fairness in the comparative experiments, the maximum number of iterations (IterMax) and the population size (PopSize) for all algorithms are unified. Furthermore, to eliminate the influence of random contingencies, each was executed 30 times (Runtimes = 30) under the same hardware conditions. The hyplgorithm is independer-parameter settings for the optimization algorithms are detailed in Table 4.

Regarding the hyper-parameter configurations of the proposed IMA, specific values—including the 50% probability for hybrid crossover, the 50% probability for hybrid mutation, the 10% maximum iteration threshold for stagnation escape, and the 10% trigger probability for the SA-based local search—were systematically determined through extensive preliminary grid-search experiments. Setting the crossover and mutation rates to a balanced 50% effectively prevents the algorithm from premature convergence in the ultra-high-dimensional search space while preserving essential genetic diversity. Furthermore, a higher SA trigger rate or a lower stagnation threshold was empirically observed to significantly inflate the computational overhead without yielding proportional cost reductions. Therefore, the parameter settings detailed in Table 4 represent the optimal empirical trade-off between global exploration capability and computational execution efficiency.

**Figure 2:**
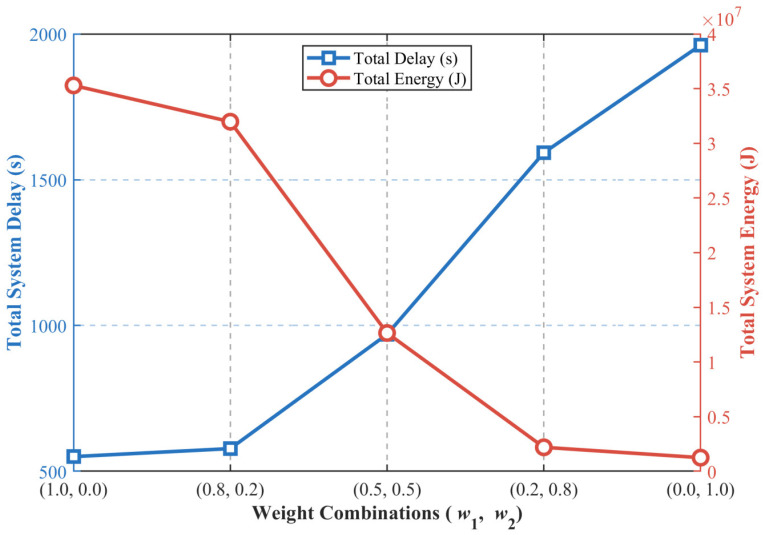
Variation in total system delay and total energy consumption under different weight combinations of latency and energy.

To ensure algorithmic robustness and reproducibility, the hyper-parameters for the IMA’s hybrid mechanisms were not selected arbitrarily; rather, they were determined through a literature-anchored calibration process. Specifically, the base swarm parameters strictly follow the foundational MA configurations [25]. To balance exploration and exploitation in the high-dimensional MINLP space, the SA cooling rate (0.95) and the targeted mutation rate (1%) were constrained within optimal theoretical bounds recommended by classical evolutionary computation literature [33,34]. Preliminary calibration within these bounds confirmed that the selected parameters yield exceptional statistical consistency (as evidenced by the zero outliers in Figure 2), rendering full-space sensitivity scanning unnecessary for this specific TIoT deployment.

### 4.2. Sensitivity Analysis of Latency-Energy Weight Coefficients

The core optimization objective of this work is to minimize the weighted sum cost of system latency and energy consumption, where the weight coefficients directly determine the optimization preference between low-latency and low-energy operation of the system. In practical district heating systems, the requirements for latency and energy consumption vary significantly across different application scenarios and deployment regions. For example, real-time valve control and fault diagnosis tasks in heating substations of urban core areas have stringent latency requirements, while terminal sensing nodes in remote suburban areas are more constrained by battery life and have stricter limits on energy consumption. Therefore, this section conducts a sensitivity analysis by adjusting the relative weight coefficients of latency and energy terms in the cost function to verify the adaptability and optimization performance of the proposed IMA under different optimization preferences, and to reveal the Pareto trade-off characteristics between system latency and energy consumption.

To ensure the single-variable principle of the experiment, all physical parameters of the thermal IoT system and hyperparameters of the optimization algorithm in this section are completely consistent with those set in Section 4.1. The physical dimension conversion constant μ defined in Equation (11) remains unchanged to avoid invalid optimization caused by the huge numerical gap between latency (in seconds) and energy consumption (in Joules). We adjust the relative weight coefficients of the total latency term and total energy term in the cost function, and set 5 groups of typical weight combinations $\left(w_{1},\;w_{2}\right)$ that satisfy $w_{1}$+$\;w_{2}$ = 1, covering the full preference range from latency-only optimization to energy-only optimization. The weight combinations are (1.0, 0.0), (0.8, 0.2), (0.5, 0.5), (0.2, 0.8) and (0.0, 1.0), respectively. The experimental results of total system delay and total energy consumption under different weight configurations are shown in Figure 2.

As can be clearly observed from Figure 2, the total system delay and total system energy consumption show a significant negative correlation Pareto trade-off relationship, which is fully consistent with the theoretical expectation of multi-objective weighted optimization, and further verifies the rationality of the established system model and the effectiveness of the IMA.

Specifically, when the weight combination is (1.0, 0.0), the optimization objective is completely focused on minimizing the system latency. The IMA preferentially selects the low-latency execution path for tasks, and the total system delay is reduced to the minimum value of 551.05 s. However, due to the lack of energy consumption constraints, the total energy consumption reaches the peak value of 3.53 × 10^7^ J. This weight configuration is suitable for mission-critical control scenarios with extremely high real-time requirements in district heating systems.

With the decrease in the latency weight w_1_ and the increase in the energy weight w_2_, the optimization objective gradually shifts to energy consumption minimization. The IMA adaptively adjusts the task offloading decision and resource allocation strategy to achieve the optimal trade-off between latency and energy consumption. For example, under the weight combination (0.8, 0.2) with latency priority, the total system delay only increases slightly to 578.51 s (an increase of 4.98% compared with the latency-only scenario), while the total energy consumption is reduced by 9.40% to 3.20 × 10^7^ J, which can achieve a good balance between real-time performance and energy saving in latency-sensitive scenarios. Under the balanced weight combination (0.5, 0.5), both the total system delay and total energy consumption are at a medium level, which can take into account the real-time control requirements of the heating system and the battery life of terminal sensing devices, and is suitable for most conventional operation scenarios of district heating networks.

When the weight combination is (0.2, 0.8) with energy priority, the total energy consumption of the system is reduced to 2.21 × 10^6^ J, while the total delay is 1592.71 s. This configuration is suitable for non-real-time data acquisition and analysis scenarios with limited terminal power supply. When the weight combination is (0.0, 1.0), the optimization objective is completely focused on minimizing the system energy consumption. The algorithm preferentially selects the low-energy execution strategy, the total energy consumption of the system drops to the lowest value of 1.27 × 10^6^ J, while the total delay reaches the peak value of 1961.75 s. This configuration is suitable for long-term periodic monitoring scenarios that are not sensitive to delay but have strict requirements on the service life of battery-powered terminal nodes.

The above sensitivity analysis results demonstrate that the proposed terminal–edge–cloud collaborative offloading model can accurately characterize the inherent trade-off between latency and energy consumption in district heating systems. Meanwhile, the proposed IMA can stably obtain the optimal scheduling solution corresponding to the optimization objective under different weight configurations, which has strong scenario adaptability and flexibility, and can meet the diversified business requirements and complex deployment scenarios in practical district heating engineering.

### 4.3. Superiority Analysis of the Terminal–Edge–Cloud Architecture

To validate the structural necessity of the proposed three-tier topology, this subsection evaluates the system performance under four distinct computation execution paradigms: Local-only, Edge-only, Cloud-only, and the proposed Collaborative architecture. By strictly constraining the boundaries of the discrete offloading decision variables within the IMA, the resource allocation optimization for each specific mode is simulated under identical high-concurrency thermal IoT conditions.

**Figure 3:**
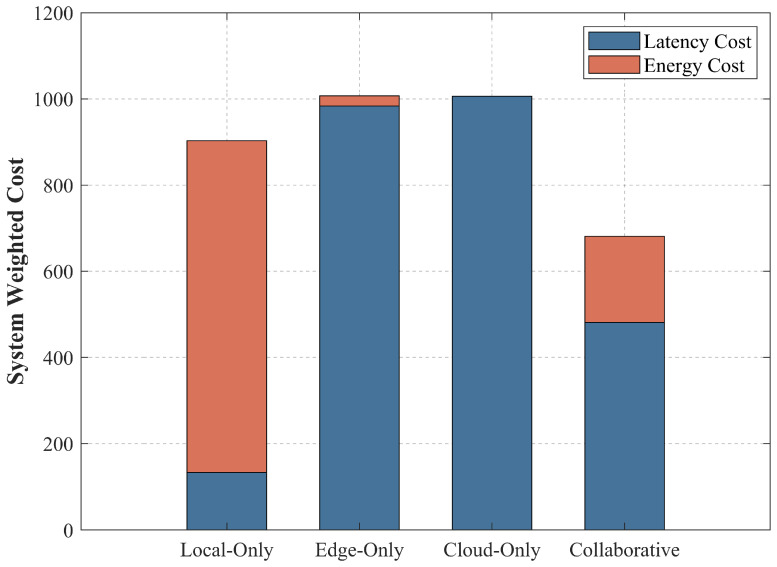
Stacked bar chart comparison of the total system weighted cost (latency and energy) among the Local-only, Edge-only, Cloud-only, and the proposed Collaborative architectures.

As illustrated in Figure 3, the stacked bar chart delineates the total system cost into latency cost and energy cost. The results indicate that each homogeneous architecture exhibits inherent structural limitations:

- Local-only Mode: The total cost is heavily dominated by the energy consumption of the terminal devices. Given the constrained inherent CPU clock frequency ($f_{local}$) of the smart sensors, executing compute-intensive tasks locally incurs substantial dynamic energy dissipation. This continuous drain accelerates the depletion of IoT node batteries, rendering it unsustainable for long-term district heating operation.
- Edge-only Mode: When tasks are exclusively offloaded to the aggregation gateways, the energy burden on the terminals is alleviated. However, this mode yields the highest overall cost, primarily driven by latency penalties. Because the edge servers possess a strictly bounded concurrent processing capacity (MaxS = 3), the sudden influx of massive concurrent tasks leads to severe queuing congestion, resulting in an exponential increase in waiting delays.
- Cloud-only Mode: Although the cloud layer provides virtually unconstrained computing power, routing massive IoT data across the Wide Area Network (WAN) incurs significant uplink transmission delays. Furthermore, the inherent fixed processing and propagation delay associated with cloud execution ($t_{colud}=\;1.8$) renders this mode inefficient for latency-sensitive thermal control tasks.

The proposed Collaborative architecture effectively mitigates the aforementioned bottlenecks, achieving the lowest total system weighted cost. Guided by the IMA, the collaborative strategy dynamically orchestrates the computational workload: it adaptively schedules tasks according to their characteristics: latency-sensitive tasks (e.g., high-frequency valve control, real-time fault detection) are offloaded to the edge to avoid WAN delays; compute-intensive tasks (e.g., calibration of indoor temperature soft-measurement models) are assigned to the cloud to prevent edge congestion; lightweight tasks (e.g., simple data filtering) are executed locally to save transmission energy. This highly adaptive offloading distribution optimizes the trade-off between energy and latency, demonstrating that the Terminal–Edge–Cloud synergy is a highly effective architectural paradigm for high-concurrency district heating networks.

### 4.4. Comparative Analysis with State-of-the-Art

**Figure 4:**
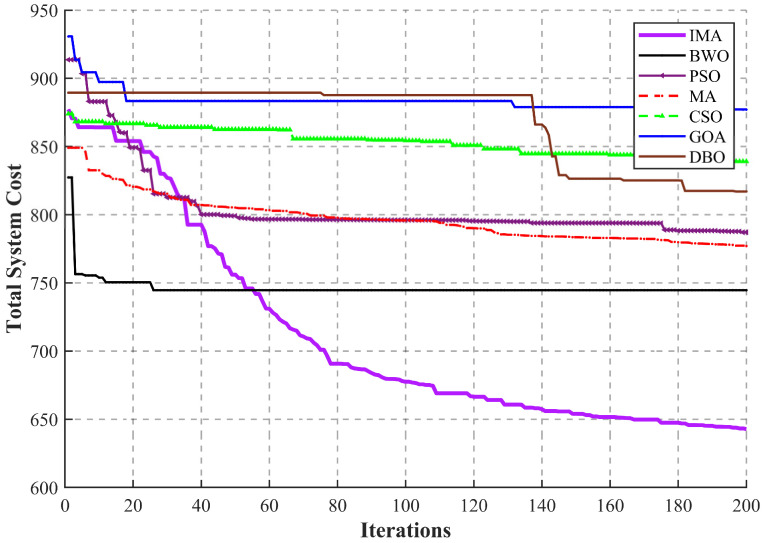
Dynamic descent trajectories of the total system weighted cost for the proposed IMA and six state-of-the-art benchmark algorithms over 200 iterations.

To comprehensively evaluate the search efficiency, optimization accuracy, and robustness against local optima, the proposed IMA is benchmarked against six state-of-the-art algorithms (BWO, PSO, MA, CSO, GOA, and DBO). The convergence behaviors of all algorithms are tested under the identical harsh high-concurrency thermal IoT environment (as defined in Section 4.1). Figure 4 illustrates the dynamic descent trajectories of the total system weighted cost over 200 iterations.

The final optimization results explicitly demonstrate the significant performance advantages of the proposed framework. The IMA achieves the lowest total system cost of 642.86, significantly outperforming BWO (744.60), MA (777.04), PSO (787.12), DBO (817.00), CSO (839.07), and GOA (877.17). Notably, compared to the standard Mayfly Algorithm (MA), the IMA reduces the total cost by approximately 17.2%, strongly validating the effectiveness of the targeted evolutionary mechanisms introduced in Section 4.

An in-depth analysis of the convergence curves reveals several critical algorithmic characteristics in tackling the highly rugged Mixed-Integer Non-Linear Programming (MINLP) space:

1. The Pitfall of Premature Convergence (The BWO Case): The Beluga Whale Optimization (BWO) algorithm (represented by the black solid line) exhibits a deceptively steep descent during the initial 20 iterations. However, this aggressive greedy exploitation strategy rapidly depletes population diversity. Consequently, BWO falls into a severe premature convergence trap, entirely losing its evolutionary momentum for the remaining 180 iterations and stagnating at a highly sub-optimal solution.
2. Stagnation in High-Dimensional Spaces (Baseline Algorithms): Algorithms such as PSO, GOA, and the standard MA experience steady initial drops but eventually plateau between costs of 770 and 880. Because the physical offloading decision involves thousands of coupled discrete and continuous variables, these baseline algorithms struggle with the “curse of dimensionality.” Lacking robust escape mechanisms, their genetic updates become ineffective when navigating the complex ridges of the fitness landscape.
3. Continuous Deep Exploration and Exploitation (The IMA Advantage): In stark contrast, the convergence trajectory of the IMA (the purple line) exhibits a highly healthy and continuous “staircase” descent. During the early iterations, the integration of the Random Position Update Mask and Differential Evolution (DE)-based Crossover effectively maintains spatial exploration diversity, preventing the algorithm from mimicking BWO’s fatal rapid convergence. More importantly, during the mid-to-late iterations (e.g., 40–80 iterations and after 100 iterations), all baseline algorithms have completely stagnated, while the IMA still maintains a stable convergence trend and continuously finds better scheduling solutions. The continuous descent trajectory in the mid-to-late iterations is closely associated with the algorithm’s ability to dynamically balance exploration and exploitation. A rigorous quantitative demonstration of how specific mechanisms (e.g., SA-driven search) independently contribute to this capability is detailed in the subsequent Ablation Study (Section 4.6). The thermal dynamic acceptance criterion enables the IMA to probabilistically tolerate inferior solutions, systematically escaping local optima traps and securing the global optimum for the Terminal–Edge–Cloud collaborative system.

### 4.5. Statistical Robustness Assessment

**Figure 5:**
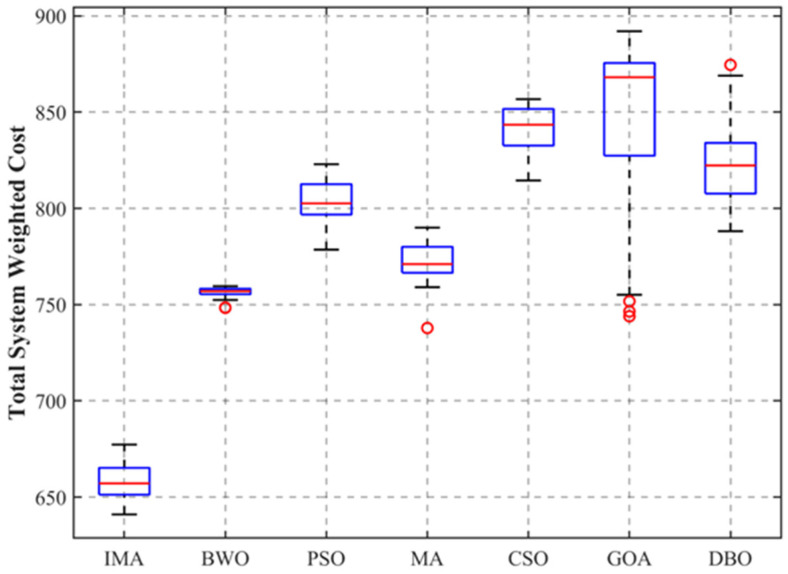
Boxplot distribution of the total system weighted cost for the proposed IMA and benchmark algorithms over 30 independent runs, illustrating statistical consistency and outliers. Blue boxes show the IQR, red lines show the median, and red circles show outliers.

Meta-heuristic optimization algorithms possess inherent stochastic characteristics due to their reliance on pseudo-random initializations. To rigorously eliminate the bias of random contingencies and comprehensively evaluate the statistical robustness of the proposed framework, all algorithms were executed independently for 30 runs under identical simulation parameters. The statistical metrics, including the Best (Minimum), Mean, Median, Standard Deviation (Std), and Worst (Maximum) values, are comprehensively evaluated. Furthermore, Figure 5 presents the distribution of the total system weighted cost via a comprehensive boxplot, visually encapsulating the interquartile range (IQR) and potential statistical outliers (red circles).

As explicitly detailed in Figure 5, the proposed IMA consistently dominates across all primary metrics. The IMA achieves the lowest Mean (658.59) and Median (657.15) costs, maintaining a strictly lower bound compared to the best-case scenarios of all other benchmark algorithms. More critically, the boxplot reveals that the IMA generates zero statistical outliers. The absence of statistical outliers and the highly compact distribution demonstrate the algorithm’s exceptional statistical consistency and insensitivity to random initialization seeds across multiple independent runs, ensuring highly predictable scheduling performance.

In contrast, baseline algorithms exhibit varying degrees of search instability. Algorithms such as GOA display a massive dispersion (Std = 46.35), indicating highly unpredictable performance. Furthermore, algorithms including MA, GOA, and DBO exhibit prominent red outlier markers, suggesting that their convergence heavily relies on serendipitous initial seeds.

It is noteworthy that while BWO presents the lowest standard deviation (2.84), this metric does not imply superior algorithmic robustness. Instead, it indicates a phenomenon of pseudo-stability caused by systematic premature convergence. As corroborated in Section 4.4, BWO rapidly loses population diversity and consistently becomes trapped in the exact same sub-optimal region (around a cost of 756) across almost all independent runs.

In contrast, the proposed IMA achieves the lowest best, mean, and median values among all comparative algorithms, and even its worst result (677.30) is significantly better than the best result of all other benchmark algorithms. Meanwhile, the IMA generates zero statistical outliers across 30 independent runs, with a small standard deviation of only 8.84, demonstrating the algorithm’s exceptional statistical consistency and insensitivity to random initialization seeds, ensuring highly predictable scheduling performance.

Ultimately, the exceptional deterministic performance of the IMA stems from the synergistic integration of the Targeted Subset Mutation and Adaptive Population Reset mechanisms. These strategies consistently preserve population diversity and guarantee a reliable escape from localized stagnation, providing the highly dependable scheduling performance required by physical district heating IoT systems.

### 4.6. Ablation Study of the Proposed IMA Mechanisms

To rigorously validate the individual contributions of the introduced algorithmic enhancements, an ablation study was conducted. While the proposed IMA incorporates multiple modifications, this section isolates the three primary driving mechanisms that govern the distinct evolutionary phases of the algorithm: early-stage exploration, mid-stage exploitation, and late-stage stagnation breakout. The ablation experiment compares the standard Mayfly Algorithm (MA) and the fully configured IMA against three functional variants: IMA-NoMask (excluding the random position update mask), IMA-NoDE (excluding the Differential Evolution-based crossover), and IMA-NoSA (excluding the Simulated Annealing-driven deep local search).

**Figure 6:**
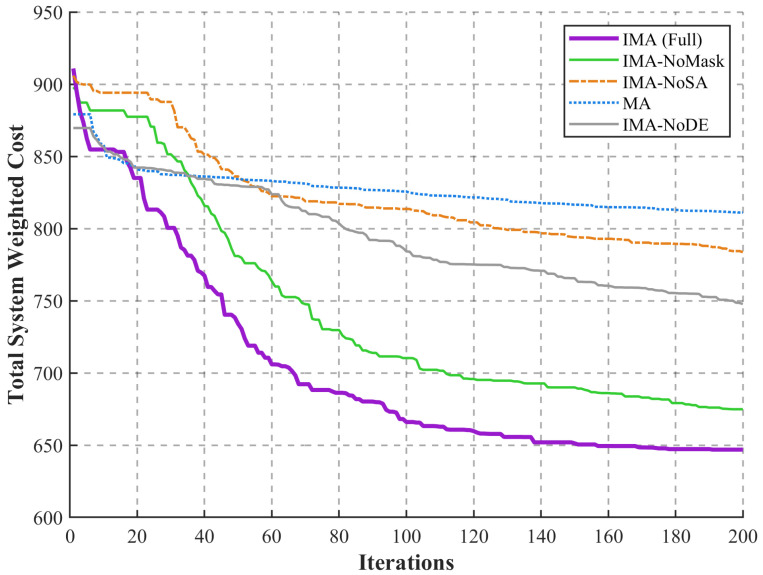
Ablation study illustrating the convergence trajectories of the Full IMA, its three functional variants and the standard MA.

Figure 6 illustrates the convergence trajectories of the Full IMA, its three functional variants (IMA-NoMask, IMA-NoDE, IMA-NoSA), and the standard MA over 200 iterations:

- The Baseline Limitation (MA): The standard MA (blue dotted line) exhibits severe premature convergence, permanently stagnating near a high cost of 811.10 after merely 20 iterations. This confirms that the canonical algorithm lacks the necessary mechanisms to navigate the highly rugged, high-dimensional MINLP landscape of the collaborative offloading problem.
- Impact of the Update Mask (IMA-NoMask): When the random position update mask is disabled (green solid line), the algorithm exhibits a noticeably delayed descent trajectory during the initial phase (iterations 0–40) compared to the Full IMA. This visual delay confirms that the mask mechanism effectively expands the initial search radius and enhances global spatial exploration, preventing early clustering.
- Impact of DE-Crossover (IMA-NoDE): The IMA-NoDE variant (gray solid line) demonstrates a sluggish descent momentum during the mid-term iterations. By reverting to the standard shuffle crossover, the algorithm fails to efficiently exchange high-quality genetic information among individuals, highlighting that the DE mutation is critical for accelerating local exploitation and maintaining a steep convergence gradient.
- Impact of SA-driven Search (IMA-NoSA): The IMA-NoSA variant (orange dash-dot line) completely loses its evolutionary momentum in the middle and later stages, flattening out prematurely around a cost of 783.51. Without the Metropolis acceptance criterion provided by the SA mechanism, the algorithm strictly rejects all inferior solutions, rendering it mathematically impossible to escape the deep local optima traps inherent to discrete resource allocation.

Ultimately, the Full IMA (purple dashed line) strictly dominates all sub-variants throughout the entire iterative process, achieving the lowest total system weighted cost. The quantitative statistical results further validate the independent contribution of each core improvement mechanism and their synergistic enhancement effects. Compared with the standard Mayfly Algorithm (MA), which achieves a total weighted cost of 811.10, the IMA-NoMask variant reduces the cost to 674.87 (a 16.80% reduction), the IMA-NoDE variant reduces the cost to 748.64 (a 7.70% reduction), and the IMA-NoSA variant reduces the cost to 783.51 (a 3.40% reduction). In contrast, the fully configured IMA achieves the optimal total cost of 646.90, corresponding to a 20.24% cost reduction over the standard MA.

More importantly, compared with the full IMA, disabling any single improvement mechanism will lead to significant performance degradation: removing the random position update mask increases the total cost by 4.32%, removing the DE-based arithmetic crossover increases the cost by 15.73%, and removing the SA-driven deep local search increases the cost by as much as 21.12%. These ablation results explicitly prove that the proposed enhancements are not mutually redundant; rather, they function synergistically to dismantle the specific optimization bottlenecks at different evolutionary stages of the algorithm.

### 4.7. Sensitivity Analysis Under Dynamic Thermal IoT Scenarios

To comprehensively evaluate the adaptability and scalability of the proposed and benchmark algorithms under high-stress industrial DHS scenarios, this section assesses system performance as the total number of concurrent tasks (N×M) expands from 100 to 750. This test range corresponds to the decision variable dimension growing up to 2250 dimensions, as each task is encoded with 3 independent decision variables (offloading decision, bandwidth allocation, and computing resource allocation).

**Figure 7:**
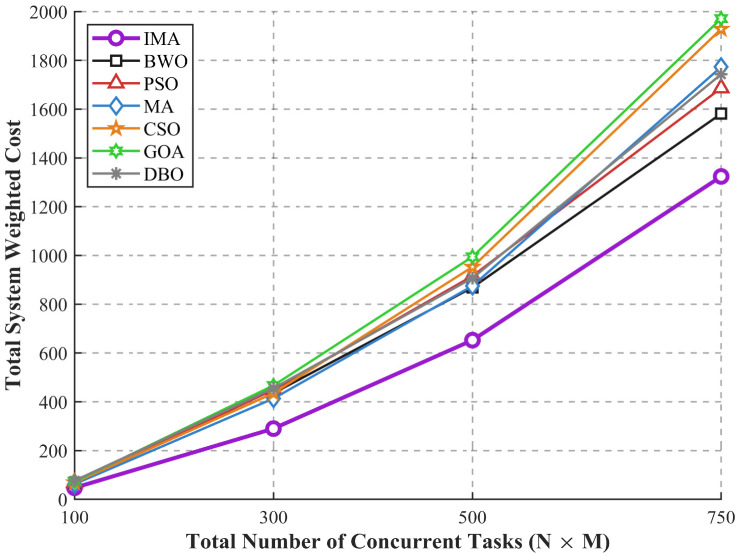
Scalability analysis of the proposed framework: cost escalation trend under varying total numbers of concurrent tasks.

Figure 7 illustrates the cost escalation trend alongside the expanding task scale. From an objective physical perspective, as the total number of concurrent tasks increases, the constrained wireless communication bandwidth, limited computational capacity of local terminal chips, and fixed edge gateway resources face severe saturation. Concurrently, the algorithmic search space encounters a significant “curse of dimensionality” as the variable dimension expands. Consequently, all evaluated algorithms exhibit a monotonic upward trajectory in total system cost as the number of concurrent tasks increases.

The distinct structural superiority of the proposed IMA is explicitly demonstrated by its gentler growth gradient compared to all benchmark algorithms. During the low-concurrency phase (N×M≤300), the performance gap between IMA and the benchmark algorithms is marginal, as the compact search space allows conventional swarm intelligence mechanisms to locate acceptable sub-optimal offloading strategies, though IMA still maintains the lowest system cost across all low-scale test cases.

Crucially, as the task scale expands to N×M=500 and 750 (the upper limit of practical DHS engineering deployment), baseline algorithms (particularly GOA and CSO) suffer from severe performance deterioration, with a steep surge in system cost approaching 2000 at the maximum test scale. Their fixed exploration and exploitation mechanisms fail to adapt to the high-dimensional, rugged MINLP landscape, leading to unbalanced resource allocation, massive task queuing, and excessive energy consumption. Even the second-best performing algorithm BWO exhibits a sharp cost increase, reaching approximately 1580 at N×M=750.

In stark contrast, the performance gap between IMA and the comparative algorithms widens significantly at these higher task scales. Driven by the robust spatial exploration of the random position update mask, the balanced search capability of the DE-based crossover, and the local optima escape ability of the SA-driven local search, IMA successfully avoids edge congestion and orchestrates efficient offloading distribution even in the 2250-dimensional search space at N×M=750. At the maximum practical engineering boundary, IMA firmly maintains its dominance with a total system cost of approximately 1330, which is 15.8% lower than the second-best BWO algorithm. This widening performance margin definitively proves the IMA’s exceptional scalability, and validates its strong potential for practical deployment in large-scale Terminal–Edge–Cloud collaborative DHS architectures.

It is worth noting that the maximum test scale of 750 concurrent tasks aligns with the physical capacity limits of practical industrial IoT deployments, where a single edge gateway typically manages no more than 150 high-frequency terminal devices to ensure stable communication reliability, avoid excessive channel interference, and prevent queuing congestion. Within all tested practical application ranges, IMA consistently maintains the best optimization performance and unparalleled scalability. For all typical heavy workloads in district heating systems, IMA remains the most robust and cost-effective optimization solution, and addressing the optimization performance under more extreme ultra-high-dimensional scenarios will be explored as a key direction in our future work.

## 5. Conclusions

Aiming at the latency and energy consumption bottlenecks of massive TIoT data processing in intelligent district heating systems, this paper proposes a terminal–edge–cloud collaborative computation offloading and resource allocation framework, and designs a tailored improved mayfly algorithm (IMA) to solve the resulting complex MINLP problem.

The experimental results demonstrate that the collaborative architecture effectively mitigates the bottlenecks inherent in traditional isolated computing paradigms by dynamically orchestrating the workload across local devices, edge servers, and cloud infrastructure. Furthermore, by incorporating advanced evolutionary mechanisms—such as a random position update mask, differential evolution-based crossover, and a simulated annealing-driven deep local search—the proposed IMA successfully overcomes the premature convergence issues typical of standard heuristic algorithms. The empirical evaluations confirmed that within practical industrial scales (scaling up to 750 concurrent tasks and a 2250-dimensional decision space), the IMA exhibits exceptional statistical robustness with zero outliers, high convergence accuracy, and superior scalability, significantly outperforming classical benchmark algorithms and achieving a 15.8% cost reduction over the most competitive baseline under maximum load.

Despite these substantial contributions, this study has two main limitations. First, in ultra-high-dimensional environments, the IMA is susceptible to “iteration starvation” under strictly bounded computational budgets, which aligns with the No Free Lunch theorem. Second, the current model primarily assumes static edge server deployments and ideal sensor reliability, without fully accounting for potential physical sensor degradation, data packet loss and channel fading in the harsh thermal environments of DHS. Therefore, future research will be carried out from the following aspects: (1) For the ultra-large-scale high-dimensional optimization problem, a distributed IMA based on edge-node-level divide-and-conquer strategy will be designed: each edge server maintains a sub-population to optimize the tasks in its coverage area, and the global optimal solution is obtained through periodic information aggregation between edge nodes, to alleviate the iteration starvation issue. (2) Considering the sensor degradation, data packet loss and channel fading in harsh thermal environments, a robust offloading model with chance constraints will be constructed, and the IMA will be optimized to adapt to uncertain scenarios to improve the engineering adaptability of the algorithm. (3) A dynamic online offloading framework based on sliding window and IMA will be designed, which can update the scheduling strategy in real time according to the time-varying task arrival and heat load changes in actual DHS operation. (4) The proposed framework will be deployed and verified in an actual district heating system, and the algorithm will be further optimized according to the field operation data to improve the engineering practicability. (5) Designing a sliding-window IMA framework for asynchronous task arrivals and incorporating chance-constrained programming to handle uncertainties like packet loss and channel fading. The current model assumes normal operation of terminal devices and edge servers. In practical DHS deployments, node failures, gateway outages, and intermittent communication interruptions may occur due to harsh thermal environments. In future work, we will extend the current framework to a reliability-aware and fault-tolerant offloading model by incorporating device failure probabilities, server availability constraints, and recovery/redundancy mechanisms into the optimization process.

In addition, recent studies on edge computing and resource allocation have shown that more sophisticated collaborative optimization strategies can further improve task execution efficiency in complex networked environments. For example, hierarchical optimization has been investigated for task execution cost minimization in D2D-assisted mobile edge computing networks, where multi-layer coordination among devices, edge nodes, and network resources is exploited to improve the global scheduling performance [35]. Meanwhile, joint trajectory, resource, and access optimization has also been studied in multi-UAV collaborative mobile edge computing systems, demonstrating the effectiveness of cross-layer coupled optimization in highly dynamic low-altitude communication environments [36]. These recent advances provide meaningful insights for our work. However, unlike D2D-assisted MEC or UAV-assisted MEC scenarios, the district heating TIoT environment considered in this paper is characterized by relatively static infrastructure, strict physical service boundaries, and strongly coupled discrete offloading decisions and continuous resource allocation variables under stepwise queuing-delay constraints. Therefore, as an important future direction, the proposed IMA framework can be further extended toward hierarchical and reliability-aware collaborative optimization by integrating multi-layer coordination, dynamic topology adaptation, and fault-tolerant scheduling mechanisms for more complex DHS deployment scenarios.

The proposed framework and algorithm provide a low-latency, energy-efficient, and highly reliable scheduling solution for the intelligent operation of modern DHS, directly supporting real-time monitoring, precise control, and intelligent diagnosis of heating networks. This work not only enriches the application of edge computing and swarm intelligence algorithms in smart energy systems but also provides technical support for the low-carbon and sustainable transformation of urban energy infrastructure.

## Figures and Tables

**Table 1:** Complete parameter index and description for the terminal–edge–cloud collaborative offloading model.

**Parameter Group**	Parameter Symbol	Index	Description	Unit
System Architecture Parameters	N	-	Number of edge servers (aggregation gateways) in the system	-
	M	-	Number of terminal devices covered by each edge server	-
	I	i∈I	Set of concurrent tasks in a single scheduling cycle, I = {1, 2, …, N × M}	-
Task Attribute Parameters	$T_{i}$	i∈I	Input data size of task i	bits
	$C_{i}$	i∈I	Required CPU computation cycles to complete task i	cycles
	$f_{\mathrm{local}}$	i∈I	Inherent CPU clock frequency of the terminal device generating task i	Hz
Decision Variables	$x_{i}$	i∈I	Discrete offloading decision variable: $x_{i}$ = 1 (local execution), $x_{i}$ = 2 (edge offloading), $x_{i}$ = 3 (cloud offloading)	-
	$u_{i}$	$i\in I_{edge}$	Proportion of uplink bandwidth allocated to task i (only valid for edge/cloud offloading tasks)	-
	$v_{i}$	$i\in I_{edge}$	Proportion of edge CPU computing resources allocated to task i (only valid for edge offloading tasks)	-
Communication Model Parameters	W	-	Total channel bandwidth of the wireless communication system	Hz
	P	-	Transmission power of terminal devices	W
	$\eta_{i}$	i∈I	Channel state information (including path loss and antenna gain) between terminal i and its associated edge server	-
	$\sigma^{2}$	-	Background noise power of the wireless channel	W
	R_i_	i∈I	Uplink transmission rate of task i	bits/s
	${rank}_{i}$	$i\in I_{edge}$	Queuing order of task i among all tasks offloaded to the same edge server	-
	$t_{wait}^{tx}$	-	Base waiting time for a single transmission round at the edge gateway	s
	$D_{tx,i}$	$i\in I_{edge}$	Total transmission delay of task i (including physical transmission time and queuing delay)	s
	$E_{tx,i}$	$i\in I_{edge}$	Transmission energy consumption of terminal device for task i	J
Computation Model Parameters	$K_{a}$	-	Dynamic energy consumption coefficient of the local terminal chip	J/cycle/Hz^2^
	$D_{local,i}$	i∈I	Computation delay of task i executed locally	s
	$E_{local,i}$	i∈I	Energy consumption of task i executed locally	J
	$C_{edge}$	-	Total CPU computing capacity of a single edge server	Hz
	$t_{\mathrm{wait}}^{cp}$	-	Base waiting time for a single computation batch at the edge server	s
	$D_{edge,i}$	$i\in I_{edge}$	Total delay of task i executed at the edge server	s
	$K_{b}$	-	Inherent energy consumption coefficient of the edge server chip	J/cycle/Hz^2^
	$E_{edge,i}$	$i\in I_{edge}$	Total energy consumption of task i executed at the edge server	J
	$t_{cloud}$	-	Fixed cloud processing delay and WAN propagation delay	s
	$D_{cloud,i}$	$i\in I_{cloud}$	Total delay of task i executed at the cloud platform	s
Objective Function Parameters	TotalDelay	-	Total system delay in a single scheduling cycle	s
	TotalEnergy	-	Total system energy consumption in a single scheduling cycle	J
	μ	-	Physical dimension conversion constant (converts Joules to kilowatt-hours), μ = 3.6 × 106	J/kWh
	$w_{1}$	-	Weight coefficient for system delay in the cost function	-
	$w_{2}$	-	Weight coefficient for system energy consumption in the cost function	-
	F	-	Weighted sum cost function of system latency and energy consumption	-

**Table 3:** Physical and network parameters of the thermal IoT system.

Parameter	Description	Value
*N*	Number of edge servers	5
*M*	Number of terminal devices per edge server	100
*MaxS*	Maximum concurrent processing capacity of an edge server	3 tasks
*T_i_*	Input data size of thermal sensing tasks	[0.5, 20] MB
*C_i_*	Required CPU computation cycles per task	[0.1, 6] × 10^9^ cycles
*f*_local_	CPU clock frequency of terminal devices	1.1 GHz
*C*_edge_	Total CPU computing capacity of edge servers	20 GHz
*t*_cloud_	Fixed processing delay in the cloud	1.8 s
*W*	Total channel bandwidth	50 MHz
*P*	Transmission power of terminal devices	0.25 W
*K_a_*	Dynamic energy consumption coefficient of local chip	10^−24^
*K_b_*	Inherent energy coefficient of edge server	10^−27^
*σ* ^2^	Background noise power	1.5 × 10^−8^ W
$t_{wait}^{tx},\;t_{wait}^{cp}$	Base waiting time for transmission and computation	0.1 s, 0.1 s
$w_{1}$, $w_{2}$	Weight coefficients for latency and energy	0.5, 0.5

**Table 4:** Hyper-parameter settings of the comparative optimization algorithms.

Parameter	Description	Value
PopSize	Population size for all algorithms	100
IterMax	Maximum number of iterations for all algorithms	200
Runtimes	Number of independent repeated runs	30
Tempinitial	Initial temperature for SA in IMA	60
CoolingRate	Temperature cooling rate for SA in IMA	0.95
Gentimes	Markov chain length for deep local search in IMA	50
µ	Targeted subset mutation rate in IMA	1% (0.01)
Threshold	Stagnation detection tolerance in IMA	10^−5^

## Data Availability

The data presented in this study are available in the article.

## Abbreviations

The following abbreviations are used in this manuscript:

BWO	Beluga whale optimization
CMOS	Complementary metal-oxide-semiconductor
CPU	Central processing unit
CSO	Chicken swarm optimization
DBO	Dung beetle optimizer
DE	Differential evolution
DHS	District heating system
GOA	Gazelle optimization algorithm
IMA	Improved mayfly algorithm
IoT	Internet of Things
MA	Mayfly algorithm
MEC	Multi-access edge computing
MINLP	Mixed-integer non-linear programming
PSO	Particle swarm optimization
SA	Simulated annealing
TIoT	Thermal Internet of Things
WAN	Wide area network
